# Deformation-Induced Martensite–Martensite Interaction in 304 Austenite Stainless Steels Subjected to Tension

**DOI:** 10.3390/ma19091802

**Published:** 2026-04-28

**Authors:** Hua Wang, Qian Liu, Bo Mao

**Affiliations:** 1 Instrumental Analysis Center, Shanghai University, Shanghai 200444, China; wangh225@shu.edu.cn; 2 School of Materials Science and Engineering, Shanghai Jiao Tong University, Shanghai 200240, China

**Keywords:** martensitic transformation, martensite-martensite interaction, microstructure, crystal structure

## Abstract

The present study aims to investigate the interaction between different martensite variants (MVs) activated in an AISI 304 austenite steel subjected to tension. Particular attention is paid to the abnormal morphologies of martensite–martensite interaction (MMI) and their possible formation mechanisms during deformation-induced martensitic transformation. The abnormal morphologies of martensite–martensite interaction (MMI) were characterized. It was revealed that MMI was accompanied by the formation of extremely incoherent interfaces. MVs can continue to grow upon impinging on each other, resulting in the morphology where one MV is crossed or totally surrounded by another. The present findings provide new insight into martensite growth behavior and variant interaction and may contribute to a better understanding of the microstructural origin of the excellent strain-hardening capability and mechanical performance of metastable austenitic steels.

## 1. Introduction

Austenitic and medium-Mn steels with low stacking fault energy have received tremendous research interest due to their excellent combination of strength and ductility associated with deformation-induced martensitic transformation (DIMT) behavior [1,2,3,4]. Such outstanding mechanical performance makes these steels particularly attractive for structural and engineering applications where high energy absorption, formability, and damage tolerance are simultaneously required. In these steels, metastable austenite transforms into martensite upon straining and provides extra ductility and strain-hardening capability [5,6,7,8]. Theoretically, 24 possible martensite variants (MVs) exist, corresponding to one parent austenite (PA) according to the well-known Kurdjumov-Sachs (KS) relationship [9,10,11,12]. However, the formation of these variants is usually not random in real deformation processes, because local stress state, crystallographic orientation, and the surrounding microstructural constraints can all influence the activation of specific variants. Previous studies have shown that some specific MVs are formed preferentially in PA and interact with each other, leading to the martensite–martensite interaction (MMI) [13,14]. The MV selection and interaction significantly affect the transformation texture, austenite reversion behavior, and mechanical properties of the steels [15,16,17,18]. For example, Ji et al. [19] reported that variant selection plays a critical role in determining the crystallographic features of multi-phase microstructures. Jiang et al. [8] and Huang et al. [20] found that controlled MV selection can be effectively exploited to achieve superior strength–ductility synergy. However, despite extensive studies being carried out to investigate the MV nucleation and selection behavior [15,21], little attention has been paid to the MMI. Specifically, it remains unclear whether MV impingement leads to mutual blocking or permits continued growth through or around the neighboring variant. Furthermore, the crystallographic conditions under which different interaction modes occur, as well as their effects on subsequent martensitic evolution, have not yet been fully clarified. A lack of understanding of this process limits further insight into MMI, variant selection, growth behavior, and the resulting plastic deformation behavior of the steel.

To address this knowledge gap, MMIs in a 304 austenite stainless steel (ASS) were investigated by performing in situ tensile tests and characterized by scanning electron microscopy (SEM) and electron backscattered diffraction (EBSD). Special attention was paid to the impingement behavior between adjacent martensite variants, their crystallographic characteristics, and the possible geometric compatibility governing their interaction. Some interesting MMI phenomena were observed, and the underlying mechanism was discussed. The present work is expected to provide new insight into the interaction and growth behavior of deformation-induced martensite in metastable austenitic steels.

## 2. Materials and Methods

**Figure 1:**
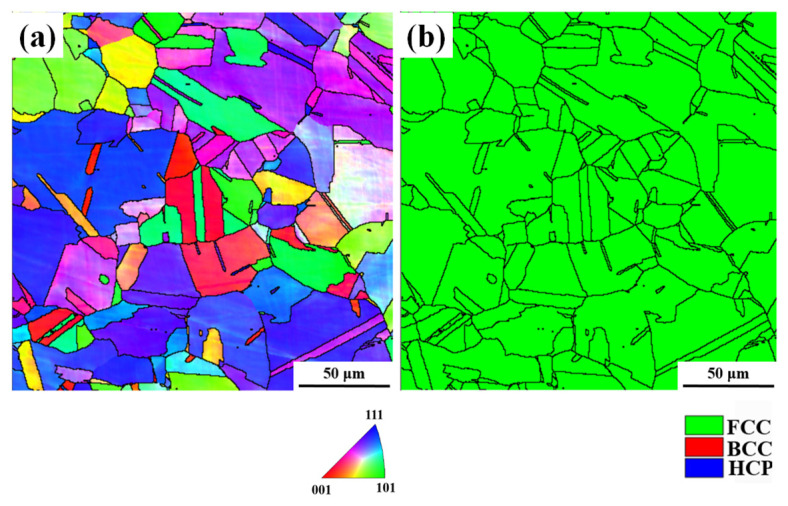
Initial microstructure of the 304 ASS after annealing: (**a**) IPF map and (**b**) phase map. The phases with face-cubic crystal structure (FCC), body-cubic crystal structure (BCC), and hexagonal close-packed crystal (HCP) structure are marked by green, red, and blue colors, respectively.

**Figure 2:**
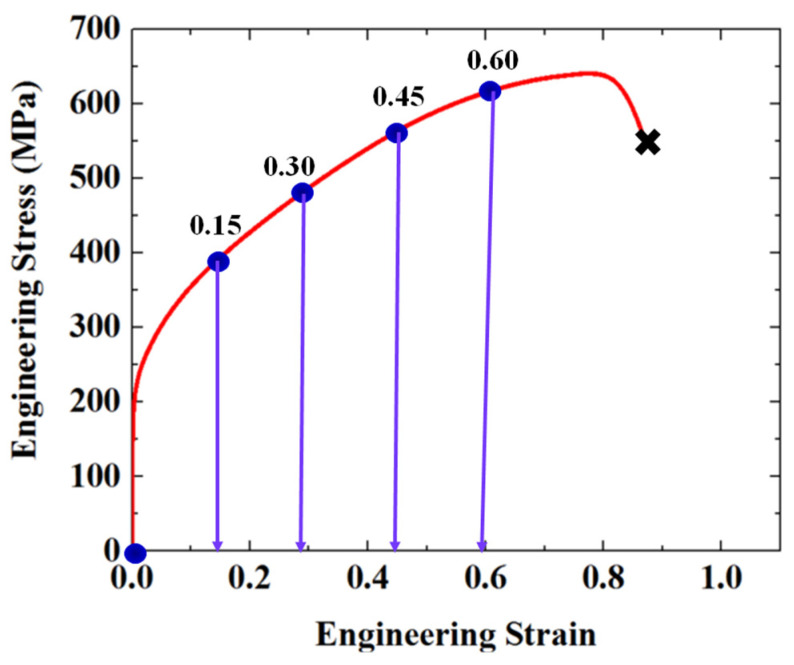
Engineering stress–strain curve of the 304 ASS during uniaxial tension.

**Figure 3:**
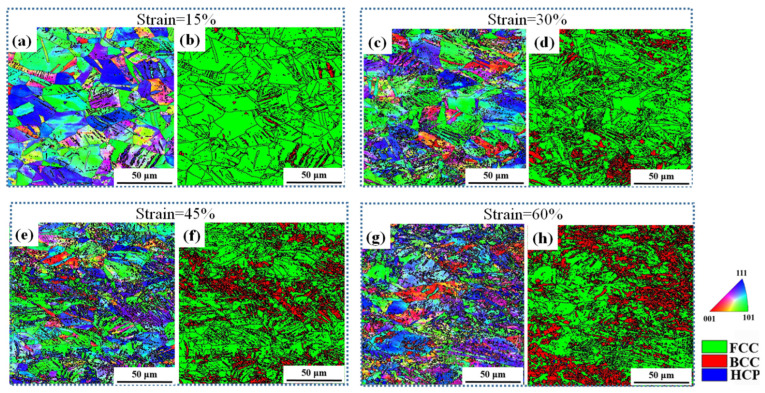
IPFs (**a**,**c**,**e**,**g**) and PMs (**b**,**d**,**f**,**h**) of the microstructure of ASS with tensile strain of (**a**,**b**) 15%, (**c**,**d**) 30%, (**e**,**f**) 45%, and (**g**,**h**) 60%.

The material used in this study is a cold-rolled commercial AISI 304 ASS block with the chemical composition of C-0.03, Mn-1.78, Cr-18.72, Ni-8.23, and Fe balance (wt.%). The block was homogenized at 1427 K in a vacuum furnace for 2 h and then water-quenched to get a full austenite microstructure, as shown in Figure 1. The homogenization treatment was conducted to reduce the effect of prior microstructural heterogeneity and to provide a relatively uniform initial microstructure for subsequent deformation analysis. Dog-bone samples were prepared according to the ASTM D638 standard [22] and subjected to uniaxial tensile tests using a 3400 machine at a strain rate of 0.001/s. In situ tensile tests were interrupted at engineering strains of 15%, 30%, 45%, and 60% for microstructural characterization, as schematically illustrated in Figure 2. A specific region of interest was identified and repeatedly examined by SEM/EBSD at each strain level, enabling continuous tracking of microstructural evolution in the same region. EBSD characterization was performed using a JEOL-2100F microscope (Tokyo, Japan), and the microstructural evolution at each strain level is presented in Figure 3. Based on the EBSD results, the crystallographic characteristics of the parent austenite and the deformation-induced martensite were analyzed, with particular attention paid to martensite variant distribution and their interaction behavior in the selected area.

## 3. Results

Figure 3 shows the microstructural evolution of the AISI 304 ASS sample after interrupted tensile tests. Inverse Pole Figure (IPF) maps and Phase Maps (PM) for each strain level are presented. At a strain of 15% (Figure 3a,b), lenticular martensite laths with BCC structure are observed within some PA grains. This indicates that martensitic transformation has already been initiated at the early stage of plastic deformation, although the transformed regions remain relatively limited in both size and number. With increasing strain, multiple MVs with distinct orientations, distinguished by different colors in the IPF maps, are activated within the same PA grains (Figure 3c–h). The appearance of differently colored regions suggests that variant selection becomes more complex as deformation proceeds, and that several crystallographically distinct MVs can nucleate and grow concurrently within individual austenite grains. Moreover, the martensite morphology evolves from lenticular to globular with increasing strain, as shown in Figure 3e–h. Such a morphological transition is likely associated with the continuous growth, impingement, and interaction of neighboring martensite variants during deformation.

**Figure 4:**
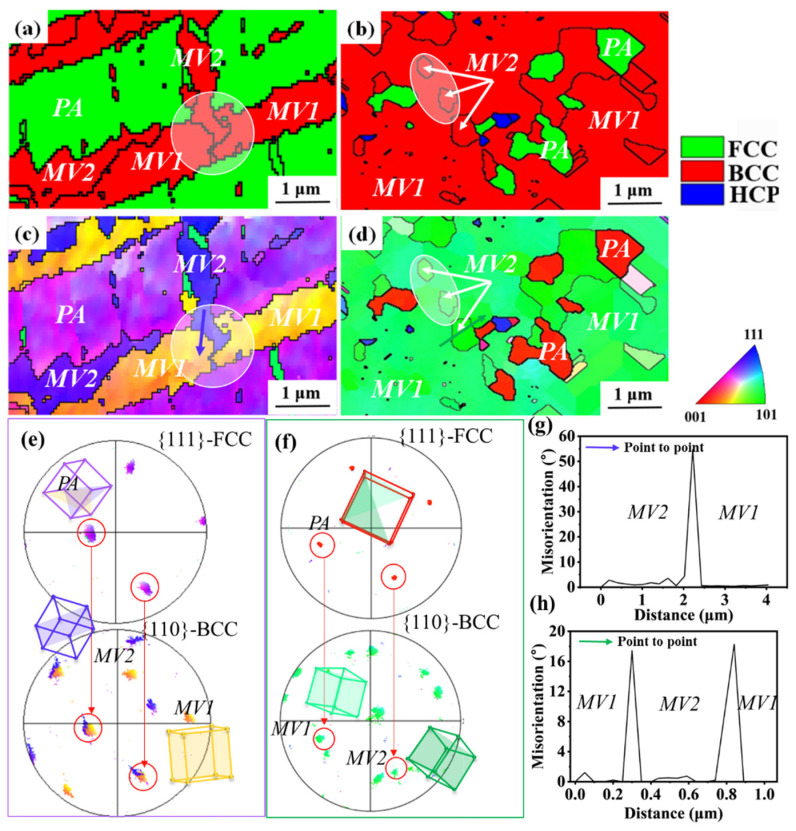
Phase map and IPFs of selected microstructure of steels at strains of (**a**,**c**,**e**,**g**) 15% and (**b**,**d**,**f**,**h**) 45%, showing the MMIs in one PA. (**a**,**b**) PMs, (**c**,**d**) IPFs, (**e**,**f**) pole figure, and 3-D unit cell of the MV1, MV2, and PA. (**g**,**h**) Point-to-point misorientation distribution.

EBSD scans with a higher resolution were performed on some selected areas containing multiple MVs to reveal their interactions. Figure 4a–d are examples extracted from the microstructure of samples at strain levels of 15% and 45%. As shown in Figure 4a,b, both microstructures contain martensite (marked in red) and the retained PA (marked in green) in the PMs. The corresponding IPFs are shown in Figure 4c and Figure 4d, respectively. Notably, two lath-shaped martensite variants in purple and yellow are observed and labeled as MV1 and MV2. The clear color contrast in the IPF maps indicates that these two regions possess distinct crystallographic orientations and therefore belong to different martensite variants. The pole figure in Figure 4e shows that the {110} planes of the MVs are parallel to the {111} planes of the PA, confirming the KS orientation relationship. Moreover, as indicated by the white circles in Figure 4a,c, MV1 is crossed by MV2 (Figure 4a,c), forming an “apparent crossing” MMI morphology. In other words, MV1 is divided and crossed by MV2. Such a morphology implies that the growth of one martensite variant is not simply arrested by the presence of another variant. This phenomenon suggests that MV2 either grows at the expense of MV1 or bypasses MV1 during the interaction.

With increasing strain, two martensite variants (colored green in Figure 4d), labeled as MV1 and MV2, are observed. Combined with the pole figure and 3D crystal orientation analysis (Figure 4f), it is revealed that these martensite blocks all satisfy the KS relationship with the PA. In addition, unlike the lath-shaped martensite at low strain, the martensite morphology at high strain exhibits an island-shaped morphology. Through the white circles in Figure 4b,d, MMI morphologies where several disconnected MV2 are completely surrounded by MV1 indicate that MV1 may outgrow MV2 and totally enclose MV2 inside. Figure 4g and Figure 4f present the “point to point” plot from the purple line in Figure 4c and the green line in Figure 4d, respectively. The results show that the misorientation between MV1 and MV2 in Figure 4c and Figure 4d is approximately 54.93°<$\overset{-}{1}\overset{-}{1}\overset{-}{1}$> and 18.23°<$\overset{-}{1}\overset{-}{2}\overset{-}{2}$>, respectively. Neither satisfies a coincident site lattice (CSL) relationship. Therefore, the interfaces of MV1/MV2 are extremely incoherent, suggesting their intensive interactions. Nevertheless, the MMI morphologies in Figure 4a–d are significantly different from the previous observations that one MV blocks the other upon interactions [19,20,23,24].

**Figure 5:**
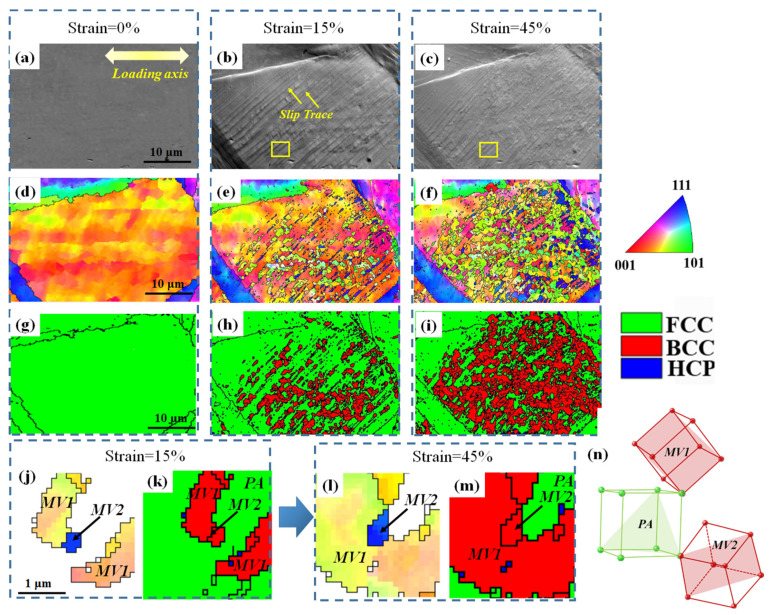
In situ observation of the MMI under tension at a strain level of 0%, 15%, and 45%. (**a**–**c**) are SEM images, (**d**–**f**) are IPFs, and (**g**–**i**) are PMs. (**j**–**m**) are IPFs and PMs corresponding to the local area marked in (**b**,**c**). (**n**) 3-D unit cells of the MV1, MV2, and PA.

**Figure 6:**
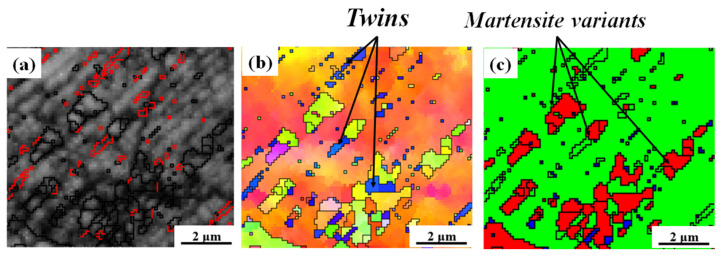
Local microstructure extracted from Figure 3e: (**a**) band contrast map. The red lines represent the Σ3 twin boundaries. (**b**) IPF showing martensite forms in the vicinity of deformation twins. (**c**) PM.

To reveal the formation process of this abnormal MMI morphology, in situ SEM and EBSD characterization were performed, and the results are shown in Figure 5. An area of interest was selected, and the undeformed state is shown in Figure 5a (SEM), Figure 5d (IPF), and Figure 5g (PM). Before deformation, the selected region exhibits a fully austenitic microstructure without detectable martensite, providing a clear initial state for subsequent comparison. With straining to 15%, the monitored microstructure undergoes a deformation-induced FCC → BCC transformation (Figure 5e,h) accompanied by the large amounts of slip traces (Figure 5b), which is attributed to the intensive dislocation activity on the {111} planes [10,11,25]. A closer check of the nucleation site of the martensite reveals that the MVs tend to form in the vicinity of the deformation twin bands (Figure 6), which has also been observed in a previous study [26,27,28].

At a strain of 15%, the martensite laths consist of isolated martensite blocks rather than forming an intact lenticular grain (Figure 5e,h). Upon increasing the strain to 45%, more martensite blocks form and intensive interactions occur (Figure 5f,i). The area enclosed by the yellow rectangle in Figure 5b,c is shown in magnified views in Figure 5j–m to illustrate the activities of MVs upon impingement. In Figure 5j,k, MV1 in the upper-left corner is in contact with MV2, whereas MV1 in the lower-right corner remains away from MV2. With increasing strain, it can be clearly seen that two patches of MV1 (Figure 5l,m) bypass MV2 and then continue to grow, merging into a larger grain upon impingement with MV2. At this stage, MV2 becomes surrounded by MV1. Moreover, the size of MV2 also slightly increases, indicating that its continuous growth after impingement is feasible. Finally, Figure 5n shows 3D unit cells of MV1, MV2, and the parent austenite (PA), along with their {110} planes (for MVs) and {111} planes (for PA), confirming that MV1 and MV2 correspond to two MVs originating from the same PA.

**Figure 7:**
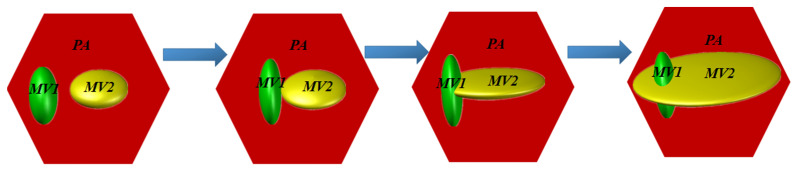
Schematic illustration of the MMI mechanism.

The anomalous MMI behavior observed in this study may shed light on the mechanism of martensite growth and provide new insight into the classical martensitic transformation theory [29]. Based on the in situ observations in Figure 5, which capture the dynamic process of one MV bypassing and surrounding another and thus provide indirect evidence for the “lateral spreading” and “growth around” behavior, a schematic illustration of the proposed MMI mechanism is presented in Figure 7. This mechanism differs from the conventional understanding that the growth of one martensite variant tends to be blocked or terminated upon impingement with another. As MV1 and MV2 are activated in PA due to DIMT, they will continue to grow upon further straining. Once they encounter each other, the growth of the two MVs will not be halted. Instead, one MV1 can spread laterally and grow around MV1, forming the “apparent crossing” and “disconnected MV islands” structure. Moreover, one salient feature that can be inferred from the observation is that the stress field of the misfit dislocations in the interfaces of MV1/PA and MV2/PA will not repel each other when two MVs approach each other, if the MV/PA interface structure follows the observation carried out by Yang et al. [25]. This possibility offers a crystallographic and interfacial basis for understanding why the two interacting variants can remain growth-capable even after contact.

The observed MMI behavior may have important implications for the mechanical properties of steel. The “lateral spreading” and “growth around” mechanisms suggest that MVs can continue to contribute to strain hardening even after impingement, potentially enhancing the sustained work-hardening capacity. The formation of “apparent crossing” and “disconnected MV islands” structures may introduce additional interfaces that act as barriers to dislocation motion, thereby affecting the strain-hardening rate and overall ductility. At the same time, these complex MMI morphologies may also influence local stress partitioning and strain accommodation within the microstructure, which could further modify the macroscopic deformation response. A follow-up investigation combining in situ mechanical testing with simultaneous microstructural characterization is planned to elucidate the structure–property correlation and validate the proposed mechanism from a mechanical behavior perspective.

## 4. Conclusions

In this study, the MMI in an ASS subjected to tension was studied by in situ tensile tests and SEM/EBSD characterization. The results demonstrate that deformation-induced martensite variants exhibit complex interaction behavior during tensile deformation, rather than simply growing independently or mutually blocking each other. The MV-MV interface is extremely incoherent, and one MV can be crossed or totally surrounded by another MV. In situ EBSD characterization confirmed that one MV can branch out by growing around another MV once impinging on each other, forming an “apparent crossing” or “disconnected MV islands” structure. A possible mechanism was proposed, and its implications were discussed.

## Data Availability

The original contributions presented in this study are included in the article. Further inquiries can be directed to the corresponding authors.
